# Visual sensitivity at the service of action control in posterior parietal cortex

**DOI:** 10.3389/fphys.2024.1408010

**Published:** 2024-05-22

**Authors:** Patrizia Fattori, Marina De Vitis, Matteo Filippini, Francesco Edoardo Vaccari, Stefano Diomedi, Michela Gamberini, Claudio Galletti

**Affiliations:** ^1^ Department of Biomedical and Neuromotor Sciences, University of Bologna, Bologna, Italy; ^2^ Institute of Cognitive Sciences and Technologies (ISTC), National Research Council (CNR), Padova, Italy

**Keywords:** vision, arm actions, affordance, object representation, action monitoring, object grasping, light influence on action, state estimator

## Abstract

The posterior parietal cortex (PPC) serves as a crucial hub for the integration of sensory with motor cues related to voluntary actions. Visual input is used in different ways along the dorsomedial and the dorsolateral visual pathways. Here we focus on the dorsomedial pathway and recognize a visual representation at the service of action control. Employing different experimental paradigms applied to behaving monkeys while single neural activity is recorded from the medial PPC (area V6A), we show how plastic visual representation can be, matching the different contexts in which the same object is proposed. We also present data on the exchange between vision and arm actions and highlight how this rich interplay can be used to weight different sensory inputs in order to monitor and correct arm actions online. Indeed, neural activity during reaching or reach-to-grasp actions can be excited or inhibited by visual information, suggesting that the visual perception of action, rather than object recognition, is the most effective factor for area V6A. Also, three-dimensional object shape is encoded dynamically by the neural population, according to the behavioral context of the monkey. Along this line, mirror neuron discharges in V6A indicate the plasticity of visual representation of the graspable objects, that changes according to the context and peaks when the object is the target of one’s own action. In other words, object encoding in V6A is a visual encoding for action.

## Introduction

The analysis of the visual form of an object is a task accomplished by the brain, relying mostly on the work of the so-called “visual cortex”, which represents a wide part of the cortical mantle of the primate brain. Since the 1980s it became evident that the task of analyzing object shape is the main goal of the visual areas of the ventral visual stream, achieving its apex in the temporal cortex (Ungerleider and Mishkin, 1982; Goodale and Milner, 1992). Since then, it was recognized that this pathway has a distinct role with respect to the dorsal visual stream, which extends to the posterior parietal cortex and forms connections with the frontal cortex, and is devoted to the computations related to transforming visual inputs into actions (Milner and Goodale, 1995). Only in this last century has it become clear that the dorsal stream is not unitary, but its job is taken over by two substreams, one more lateral (the dorso-lateral visual stream) and one more medial (the dorso-medial visual stream), both devoted to transforming visual inputs into actions (Galletti et al., 2003; Rizzolatti and Matelli, 2003).

Here we provide an overview of studies showing the kind of visual object analysis performed by neurons in an area of the dorso-medial visual stream, namely area V6A (Galletti et al., 1999b; Galletti et al., 2003), which bridges visual and somatosensory information to control actions involving eyes and arms, both in humans and non-human primates (Gamberini et al., 2021; Sulpizio et al., 2023).

## Sensory properties of the posterior parietal cortex

**FIGURE 1:**
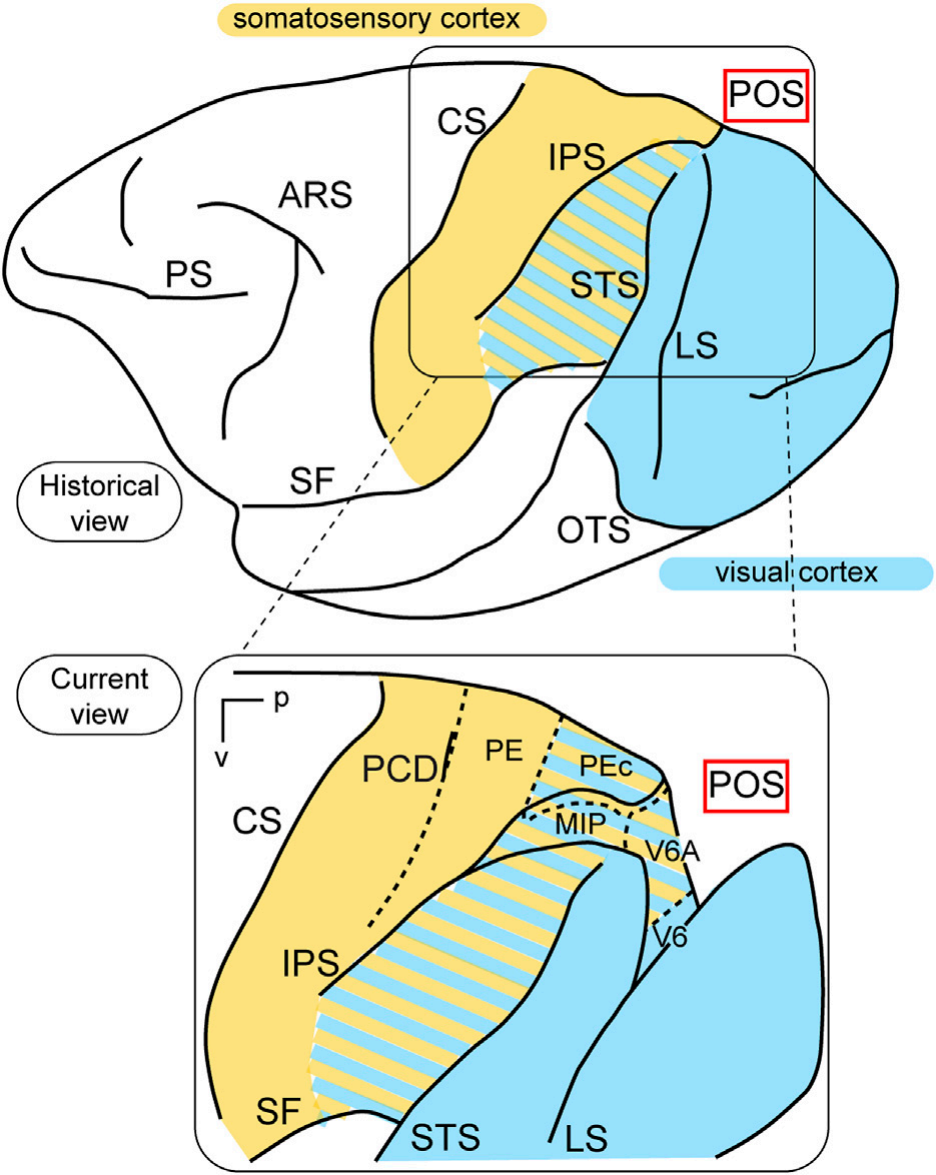
Visual functions of PPC. Top: Lateral view of a macaque’s left hemisphere. The visual cortex is colored in light blue and the somatosensory cortex in yellow. The cortex of the inferior parietal lobule containing visual as well as somatosensory cells is filled with mixed light blue and yellow lines. Abbreviations: ARS arcuate sulcus, CS central sulcus, PS principal sulcus, SF Sylvian fissure, IPS intraparietal sulcus, LS lunate sulcus, STS superior temporal sulcus, OTS occipito-temporal sulcus, POS parieto-occipital sulcus. Bottom: enlargement over the medial parietal cortex and opening of parieto-occipital sulcus highlights the location of areas V6A and V6, which are hidden within the depth of the parieto-occipital sulcus. Colored lines delimit areas with bimodal visual and somatosensory properties. Other abbreviations: p posterior, v ventral, PCD post central dimple, PE, PEc, MIP, V6A, V6 areas PE, PEc, MIP, V6A, V6.

In terms of function, the posterior parietal cortex (PPC) was considered a “terra incognita” until the 1970s. At that time, the dorsal sector of the PPC, located in the superior parietal lobule (the Brodmann’s area 5 in the macaque monkey, Brodmann, 1909) was investigated for its somatosensory properties, considering its adjoining position in relation to the primary somatosensory cortex (Brodmann’s areas 3, 1, and 2). In a pioneering study, Vernon Mountcastle and collaborators (Mountcastle et al., 1975) found neurons with passive somatosensory responses in Brodmann’s area 5, as well as neurons that were activated when the monkey performed active arm movements. In the lateral sector of the PPC, however, in Brodmann’s area 7, located in the inferior parietal lobule, they found neurons activated by visual sensory stimuli. In the same years, Hyvarinen and collaborators confirmed the presence of both visual and somatosensory responses in the lateral PPC (Hyvärinen, 1982). These studies, together, ascribed visual and somatosensory roles to the lateral PPC, and only somatosensory motor-related roles to the medial PPC, as sketched in the top part of Figure 1.

**FIGURE 2:**
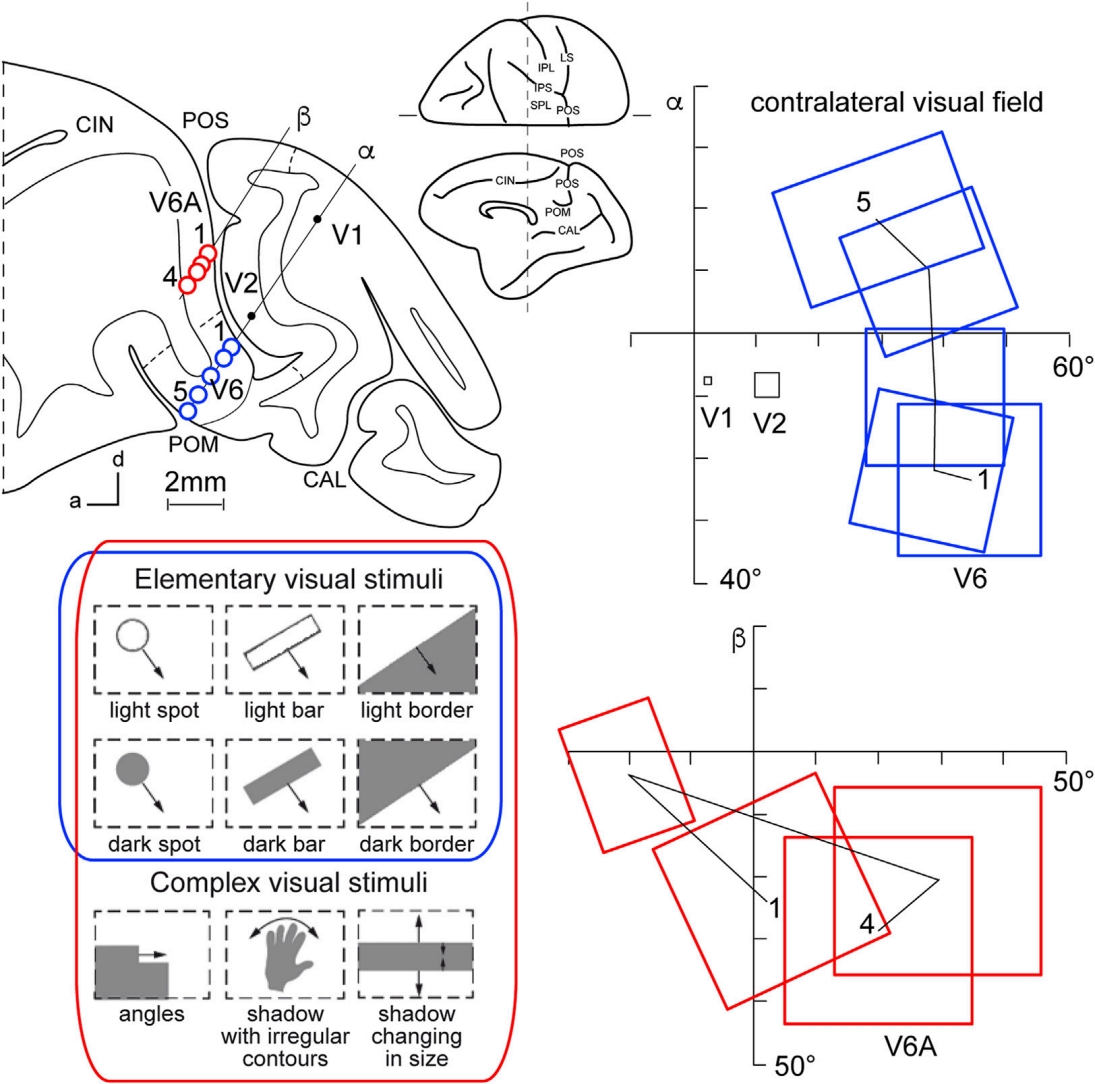
Microelectrode penetrations through the cortex of POS. Top left, Parasagittal section of the brain of *Macaca fascicularis*, taken at the level shown on the dorsal view of the brain reported at the center of the figure. Letters 'α' and 'β' on the section indicate the reconstructions of two microelectrode penetrations passing through the occipital pole and reaching the cortex of POS in the depth. Blue circles indicate V6 recording sites, red circles indicate V6A recording sites. Dashed lines on the gray matter mark the limits between different cortical visual areas. At the bottom, types of visual stimuli used in the visual test. Elementary visual stimuli, such as light/dark spots, bars, or luminous borders, and complex visual stimuli, such as dark shadows with irregular shapes and/or continuously changing in size, were moved with different directions and speeds. Elementary visual stimuli drive all visual responses in V6 but only a part of the neurons in V6A. Complex visual stimuli (bottom line) are needed to activate most of V6A’s visual responses. Right: reconstructions of receptive field sequences encountered along penetrations α (V6, blue data) and β (V6A, red data) are reported. A continuous line joins the receptive field centers of cells recorded from the same area, showing a clear topographical order in area V6 and a scattered sequence in area V6A. The numbers along these lines indicate the first and the last receptive field encountered along each cortical area. Abbreviations: CIN cungulate sulcus, POM medial parieto-occipital sulcus, V1, V2, areas V1, V2. Other details and abbreviations as in Figure 1. Adapted from Galletti et al. (1999a; 1999b) with permission from Blackwell Publishing.

This view started to change at the end of the 1980s, following a series of neuroanatomical (Covey et al., 1982; Zeki, 1986) and electrophysiological (Colby et al., 1988) studies. Colby and coworkers, in particular, recorded neuronal activity in anaesthetized monkeys from the very medial and posterior sectors of PPC, in the cortex hidden in the depth of the parieto-occipital sulcus. They showed that this PPC region is the recipient of visual inputs from many cortical visual areas and shows clear visual responses. A couple of years later, Galletti and collaborators found visual responses in the anterior bank of the parieto-occipital sulcus (the caudal part of precuneate cortex) of awake macaques (Galletti et al., 1991), and described two new visual regions that they called V6 and V6A (Galletti et al., 1990). This new knowledge regarding these medial PPC areas allowed for a reinterpretation of the maps of medial PPC with multiple bimodal areas (visual-somatosensory, PEc, MIP, V6A areas), as shown at the bottom part of Figure 1 (Colby and Duhamel, 1991; Breveglieri et al., 2008; Prevosto et al., 2011; Gamberini et al., 2018). Here, with the partial opening of the parieto-occipital sulcus, it can be appreciated that areas V6 and V6A lie on the verge of purely visual areas and bimodal visual and somatosensory areas (see Figure 1, bottom). Later on, it became clear that area V6 was a typical extrastriate visual area, with a well-defined retinotopic organization (Galletti et al., 1999a) (Figure 2, blue data), like that of adjoining areas V2 and V3. During microelectrode penetrations in area V6, the receptive fields change position continuously in the visual field in a very ordered manner, following the topographic changes in the position of the electrode tip (Figure 2, blue data). The central 20° of the visual field is represented laterally and the periphery is represented medially in the deepest parts of the parieto-occipital sulcus, as shown by recording number 5 in Figure 2. Area V6 represents the whole contralateral visual hemifield.

Area V6A shows a majority of visual neurons, but also, differently from V6, a consistent proportion of neurons that are unresponsive to visual stimulations (Galletti et al., 1999b). This functional difference mimics the difference in cytoarchitecture of the two areas, with V6 showing features typical of occipital cytoarchitecture and V6A displaying features typical of parietal cytoarchitecture (Luppino et al., 2005). V6A visual responses are poorly driven by the stimuli that are able to activate visual responses in V6, such as single borders, bars, or spots of light. The majority of V6A visual neurons need more complex visual stimuli to be activated (shown at the bottom left of Figure 2, red data), for example, complex shapes such as corners, or stimuli that continuously change orientation, velocity, or size (Gamberini et al., 2018). Lastly, area V6A does not show a clear retinotopic organization. Often, the receptive field of successive recording sites in the same microelectrode penetration were located in different portions of the visual field, sometimes in different quadrants or hemifields (Figure 2, red data) (Galletti et al., 1999b).

V6A mainly represents the contralateral part of the visual field, in particular the lower visual field, together with the medial part of the ipsilateral visual field (Galletti et al., 1999b; Gamberini et al., 2018). Interestingly, V6A shows a higher representation of the lower visual field, which is advantageous for visuomotor interactions because it is where our actions predominantly occur. The emphasis on the lower visual field supports the proposed functional role of V6A in controlling prehension (Fattori et al., 2017; Galletti and Fattori, 2018). Imaging experiments support this view (Rossit et al., 2013; Maltempo et al., 2021), and very recent experiments suggest that there is a lower visual field advantage for affordances (Warman et al., 2023), a property that is able to modulate V6A activity (Breveglieri et al., 2015); this concept will be treated later on in the sixth chapter of this review.

## Reaching and grasping properties of the medial posterior parietal cortex

Area V6A is a parietal area (Luppino et al., 2005) which occupies a (medial) part of Brodmann’s area 7 (Gamberini et al., 2020). It hosts visual and somatosensory neurons as well as neurons driven by arm movements, more strongly by active arm movements performed by the monkey (Galletti et al., 1997; Gamberini et al., 2011; Gamberini et al., 2018; Fattori et al., 2017). V6A neural activity is strongly driven by reaches performed toward visual targets (Fattori et al., 2001) and its neurons often show a clear spatial tuning (Fattori et al., 2005). Reaching activity was found in darkness, so this tuning cannot be ascribed to any kind of visual stimulation. The onset of the earliest neural discharges during reaching was not compatible with passive somatosensory stimulation, because they preceded even the earliest EMG activity (Fattori et al., 2005). These discharges were ascribed to corollary discharges of motor commands, likely to be originated by the frontal cortex (Fattori et al., 2005), which is directly connected with V6A (Galletti et al., 2004; Gamberini et al., 2009).

**FIGURE 3:**
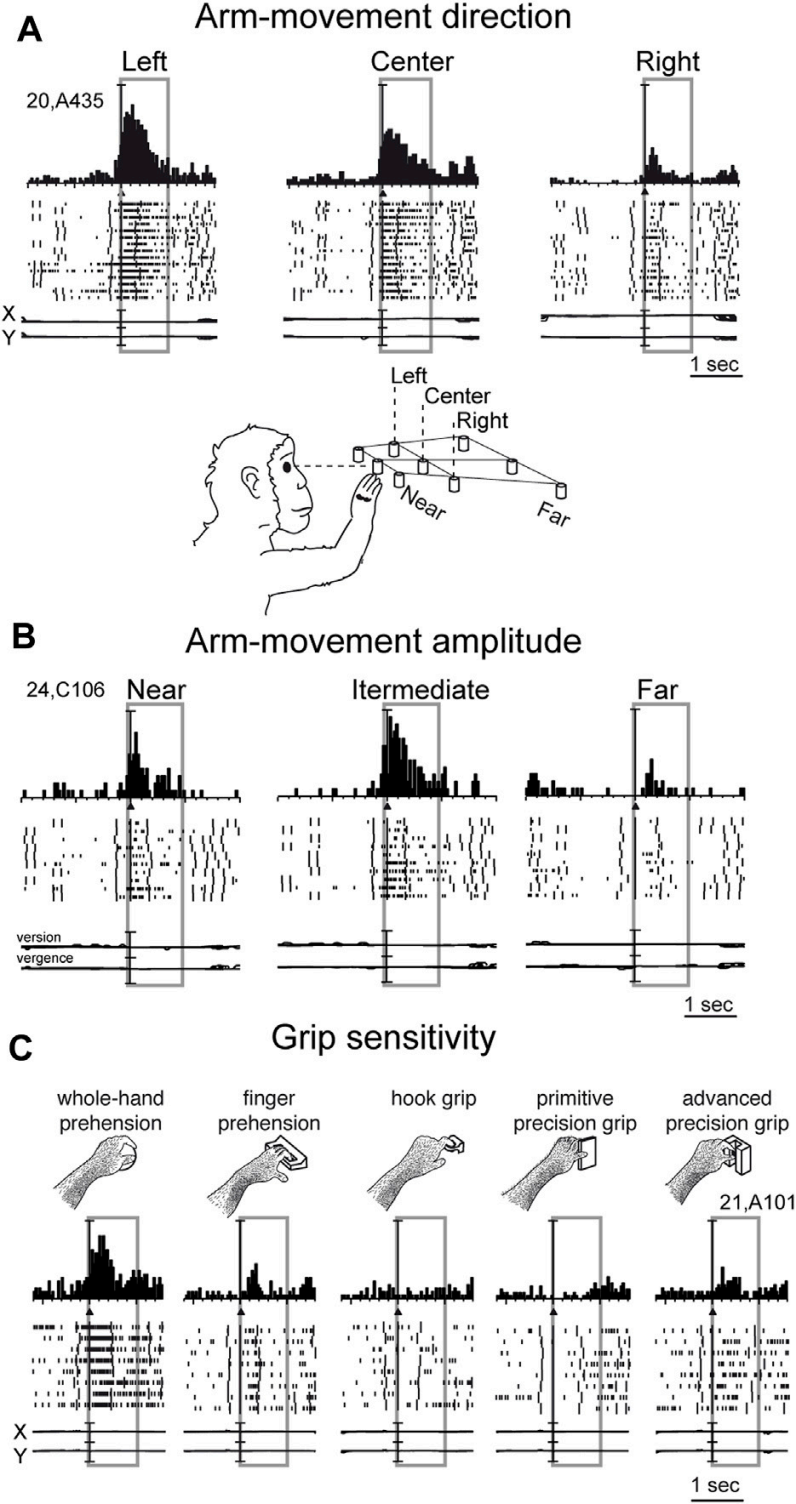
Example V6A neurons showing arm movement modulation. **(A)** Cell tuned by direction of arm reaching movement. It has an evident preference for reaches directed to the left target, with intermediate discharges for reaches to the center, and almost no discharge at all for reaches to the right. **(B)** Cell modulated by depth of arm reaching movement. It shows a clear peak of activation during reaching occurrence and a clear preference for reaches directed to the target in the intermediate position; the farthest target evoked weaker activations during reaching. **(C)** Cell modulated by grasping movements. In each panel, cell responses are shown as peri-event time histograms together with raster displays of impulse activity, aligned with the arm movement onset (black triangle). Long vertical ticks in raster displays are behavioral markers. Below cell responses, recordings of X and Y components of eye positions are reported in A and C, and recording of version and vergence are reported in **(B)**. Reaching and reach-to-grasp arm movements were performed in the dark during steady fixation. Between panels A and B a drawing shows the arm of the monkey reaching the closest central target. The gray boxes highlight movement-related activity (from movement onset to 1 s after it). In C, grasping neuron: types of grip used by the monkeys to grasp the different objects are shown. The 5 different objects are shown, one at a time, in the same spatial location as the central closest target of the reaching apparatus. Scale in A, vertical bar on histograms = 65 spikes/s; eye traces = 60°/division. In B, vertical bar on histograms = 30 spikes/s; eye traces = version 60°/division; vergence 0°–20°. In C vertical bar on SDFs = 70 spikes/s; eye traces = 60°/division. Adapted from Fattori et al. (2010), licensed under CC BY-NC-SA 4.0; Hadjidimitrakis et al. (2014b), with permission from Oxford University Press.

Reaching activity of V6A neurons is modulated by both direction (Figure 3A) and amplitude (Figure 3B) of arm movement. In other words, reaching activity encodes the movement of the arm in the 3D space around the animal. While many studies have reported modulation in the PPC by direction of arm movement (Kalaska et al., 1983; MacKay, 1992; Johnson and Ferraina, 1996; Ferraina et al., 1997; Scott et al., 1997; Snyder et al., 1997; Nakamura et al., 1999; Battaglia-Mayer et al., 2000; Battaglia-Mayer et al., 2003; Scherberger and Andersen, 2007; Chang and Snyder, 2012; Li et al., 2022), modulation by amplitude of movement has been scarcely addressed in the literature. This may be because reaching tasks have been typically studied using center-out tasks in which, being performed on a 2-D surface in front of the animal, the depth of movement remains constant across different spatial positions. The encoding of reaches bringing the arm at different distances from the body in PPC was first documented in the in Brodmann’s area 5 (Lacquaniti et al., 1995). Later on it was reported in other medial PPC areas such as V6A (Hadjidimitrakis et al., 2014a; Hadjidimitrakis et al., 2017; Hadjidimitrakis et al., 2020; Bosco et al., 2016; Bosco et al., 2019; Diomedi et al., 2020) but also neighboring areas such as PEc and PE (Hadjidimitrakis et al., 2015; Piserchia et al., 2017; De Vitis et al., 2019; Diomedi et al., 2021; Vaccari et al., 2024). Interestingly, while in the anterior part of PPC (area PE) direction and amplitude tend to be encoded by different neurons, in the posterior part of PPC (area PEc, V6A) single neurons encode both direction and amplitude of arm movement (De Vitis et al., 2019; Hadjidimitrakis et al., 2022).

While the modulation of V6A activity by direction and amplitude of reaching arm movement was in line with the established functions of the dorsomedial visual stream, as depicted in textbooks of neuroscience (e.g. Kandel et al., 2014), the presence in the dorsomedial visual stream of neural modulations tuned for the grip performed by the monkey while approaching and grasping an object was completely unexpected (Fattori et al., 2010).

In the example neuron shown in Figure 3C, the monkey hand always landed in a constant space location where different objects were presented, one at a time. Here, we see an increase in neural spike trains any time the arm action was performed, with an evident peak around the movement onset. But this movement was not a mere reaching, but rather a reach-to grasp action. Indeed, the monkey started the hand movement as in the reaching task, with the hand close to the body, and ended the movement away from the body. But now the task required the monkey to grasp and pull an object. To accomplish the task, the animal had to shape its hand, so as to form the right grip to grasp the object, pull it, and hold it in the pulled position for a fixed time before releasing the object. This task was used to study the neurons of the dorsolateral visual stream (Taira et al., 1990; Sakata, 1992). Many neurons there, in particular in an area called AIP (anterior intraparietal area; Murata et al., 2000), were found to be very sensitive to the type of grip. Surprisingly, in the dorsomedial visual stream, specifically in area V6A, most of the cells were also tuned for the type of grip, in a similar way to the example shown in Figure 3C. Here, again, as for the reaching movements, the arm actions were performed in the dark to prevent potential visual distractors from confounding the interpretation of these motor-related discharges. This example neuron was maximally responsive to the whole hand grip (left panel), which is the most rudimentary grip, while it showed lower activation when grasping a handle (finger prehension). More skilled grips, such as the hook grip (central panel) and the primitive precision grip (fourth panel), did not evoke any response, while the most skilled grip, the advanced precision grip (last panel), evoked a good peak of activation. Of course, each V6A cell showed its own grip selectivity, with different preferences for each individual grip type (Fattori et al., 2012; Breveglieri et al., 2016; Breveglieri et al., 2018). These data parallel those found in the dorsolateral visual stream (Taira et al., 1990; Jeannerod et al., 1995; Gardner et al., 1999; Gardner et al., 2007; Schaffelhofer and Scherberger, 2016).

## Interactions between vision and arm signals in parietal area V6A

In the previous chapters, we summarized the visual and arm movement-related properties of the medial posterior parietal area V6A. Reaching and grasping activities of V6A neurons were tested in the dark, i.e., without any visual confounding, either from the moving limb or from the environment. Under our experimental conditions, only the very small fixation point emitted light, albeit with an extremely low luminance (Fattori et al., 2001) making it insufficient for discerning details in the darkened environment. Here we show the effects of interaction between arm movement-related signals and visual signals. V6A neurons were recorded while monkeys executed reaching (Bosco et al., 2010) or reach-to-grasp movements (Breveglieri et al., 2016; Breveglieri et al., 2018) in two separate conditions: in the dark, where only the reaching target was visible, and in the light, where the monkey saw its own moving arm and the environment. As expected, the sight of the moving arm often changed the cell response to arm movements dramatically, but, while we were expecting the neural discharge to increase in the light, this was actually not always the case. For some neurons, arm movement-related modulations were stronger in the light than in the dark, but in others the light inhibited reaching activity.

**FIGURE 4:**
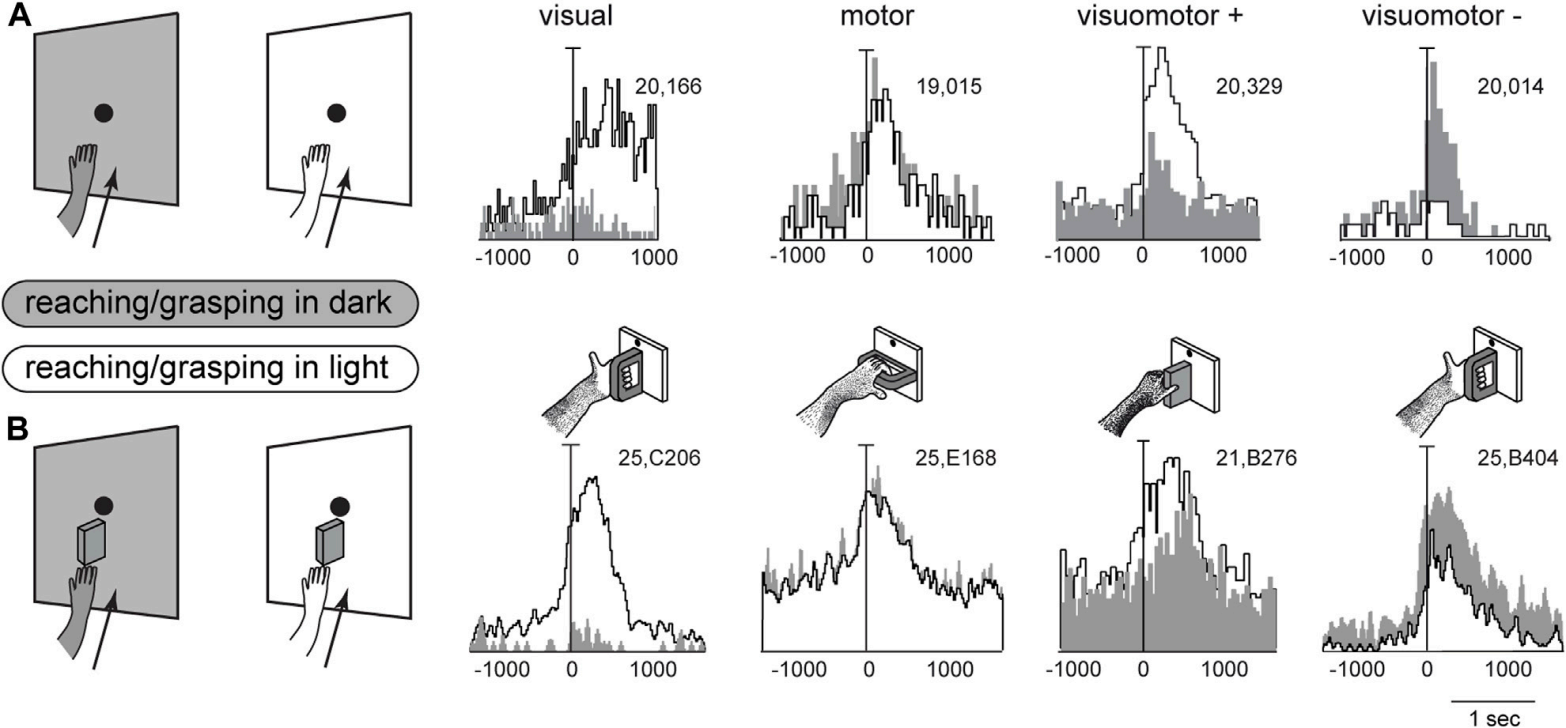
Interaction between vision and arm signals in V6A. **(A)**, First panel: visual neuron, not influenced by motor-related signals for reaching, but sensitive only to the visual feedback from the moving arm. Scale: Vertical bar on histograms, 66 spikes/s. Second panel: reaching neuron not affected by the availability of visual information. Scale: Vertical bar on histograms, 60 spikes/s. Third panel, reaching neuron more activated in the light than in the dark. Scale: Vertical bar on histograms, 185 spikes/s. Right, reaching neuron activated only in the dark. Scale: Vertical bar on histograms, 55 spikes/s. **(B)**, First panel: visual neuron, not influenced by motor-related signals for grasping, but sensitive only to the visual feedback from the moving hand and its interaction with the object. Scale: Vertical bar on histograms, 85 spikes/s. Second panel: grasping neuron not affected by the availability of visual information. Scale: Vertical bar on histograms, 45 spikes/s. Third panel, grasping neuron more activated in the light than in the dark. Scale: Vertical bar on histograms, 118 spikes/s. Right, grasping neuron activated only in the dark. Scale: Vertical bar on histograms, 80 spikes/s. On the right, cartoons of the Reach and Reach-to-Grasp tasks in the dark (gray) and in the light (white) conditions are shown. Other details as in Figure 3. Adapted from Bosco et al. (2010), licensed under CC BY-NC-SA 4.0; Breveglieri et al. (2018), with permission from Oxford University Press.

Figure 4 shows example neurons tested in the reaching and grasping task in dark and light conditions. In Figure 4A *neurons responsive for reaching* are shown. The first neuron in Figure 4A is weakly activated (if at all) by reaching in the dark, but is very strongly activated by reaching in the light. This means that it receives a strong visual input and a very weak (if any) somatosensory/somatomotor input related to the arm movement. The other three neurons in Figure 4A show motor related activity, since they discharge when the action is performed in the dark, in the absence of any visual feedback. Their activity is differently modulated by the light environment. The second neuron is almost equally activated by reaches performed in the two conditions. This indicates that the neuron does not receive any visual input. Its activity is only modulated by the somatosensory/somatomotor activity related to the arm movement. According to the classification of parietal neurons by Murata and coworkers (Murata et al., 2000), this cell type is classified as a “motor” neuron. The last two neurons in Figure 4A show different neural activity in the light and in the dark. This indicates that they do receive visual information in addition to arm movement-related information. They are classified as “visuomotor” neurons. The first neuron increases its activity in the light, i.e., the visual input enhances motor-related discharges (“visuomotor +”). The second cell strongly decreases its activity in the light, i.e., its reaching activity is reduced by visual information (“visuomotor –”). These diverse interactions between movement-related activity and vision show that there is a complex interplay between vision and reaching movement in V6A, most likely at the service of action monitoring.

Figure 4B shows four example V6A neurons tested in a *reach-to-grasp task*, with and without visual feedback. They parallel the diverse interplay between vision and arm movement-related discharges observed in Figure 4A, but in this case the movement-related discharges regard the performed grip type (Breveglieri et al., 2018). Like the above neurons that were tested during reaching, in Figure 4B parallel neural encoding is found for grasping: the first cell receives only visual inputs, the second only motor-related inputs; the other 2 cells integrate motor-related and visual-related information, with an additive effect (central cell) or a reduction (right cell) of the motor-related discharge when grasping occurs in the light.

We can assume that movement-related activity in the light condition, both for reaching and for grasping may reflect motor efferent copy, and proprioceptive and visual afferent feedbacks, whereas activity in the dark condition would reflect only the motor efferent copy and proprioceptive feedback (Fattori et al., 2017). The diverse neuronal properties shown by the example neurons in Figure 4 probably represent differences in the degree of influence exerted by visual feedback control as opposed to feedforward movement planning (Kawato, 1999; Scott, 2004; Shadmehr and Krakauer, 2008; Grafton, 2010; Shadmehr et al., 2010), encompassing both reaching and reach-to-grasp actions. This complex interplay between vision and arm-movement signals in V6A may represent a bridge from single neurons to computational models, suggesting task-dependent reweighting of sensory signals while action unfolds for the purpose of monitoring and correcting reaching and grasping actions.

## Visual encoding of object shape

The assessment of visual sensitivity in V6A was performed by projecting 2D shapes onto opaque screens (Figure 2). Later on, real 3D objects of different shapes were also tested as possible visual stimuli for this arm movement-related area.

**FIGURE 5:**
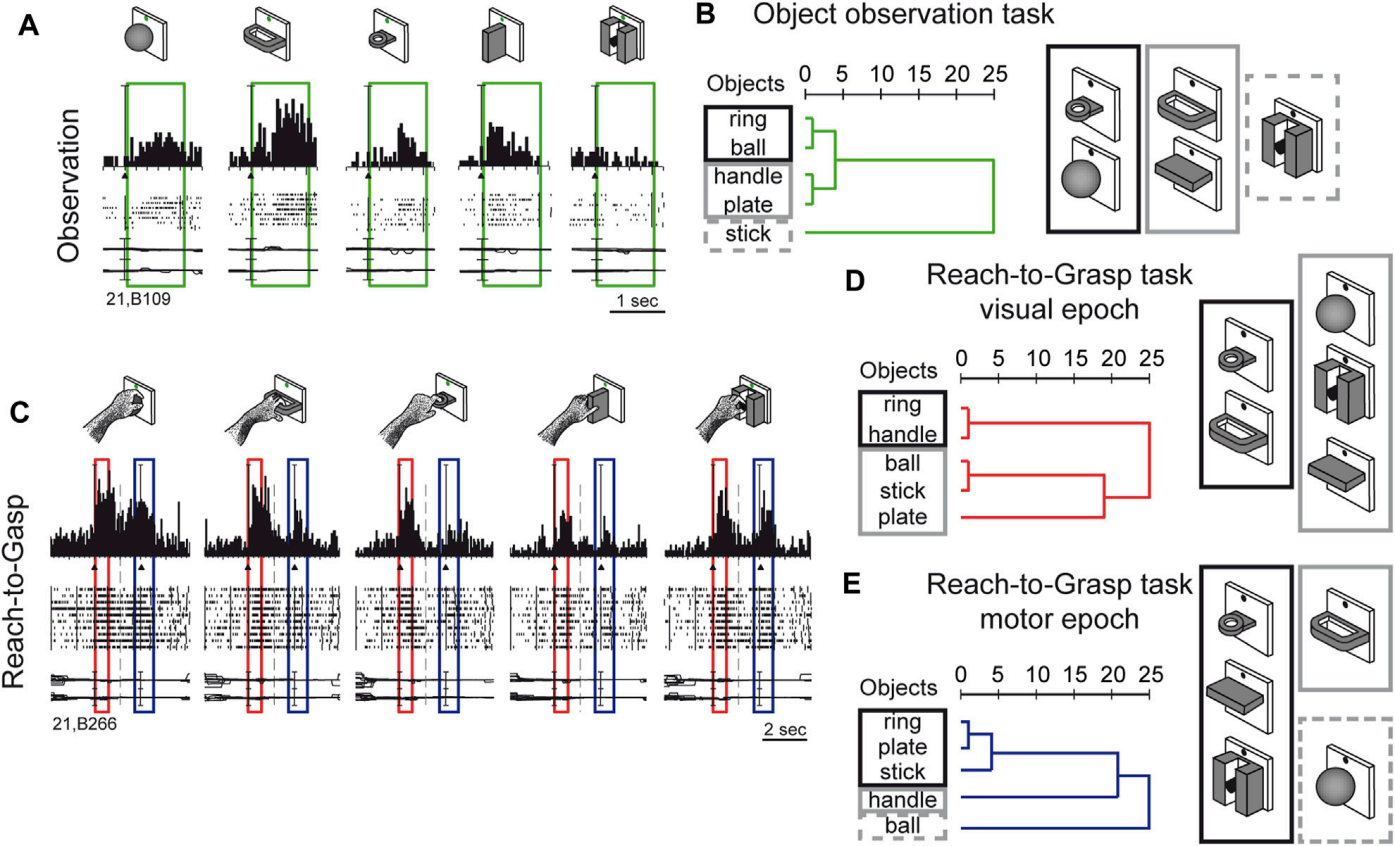
Complex encoding of object in V6A. **(A)** Neural activity of an example V6A neuron during object observation. The green boxes indicate the time of object presentation. Vertical scale on histogram: 60 spikes/s. **(B)** visual encoding in the object observation task. Dendrograms illustrating the results of the hierarchical cluster analysis in the object observation tasks. Horizontal axis in the dendrogram indicates the distance coefficients at each step of the hierarchical clustering solution. Actual distances have been rescaled to the 0–25 range. **(C)** Neural activity of an example V6A neuron during a reach-to-grasp task. Boxes indicate the time of object presentation (red) and reach-to-grasp execution (blue). Activity has been aligned twice, to object presentation and to movement onset. Cell discharges during object vision and Reach-to-Grasp execution and the visual responses are tuned for objects and the motor discharges are tuned by the different grips, displaying specific preference. Vertical scale on histogram: 80 spikes/s. Other details as in Figure 3. **(D, E)**: Dendrograms illustrating the results of the hierarchical cluster analysis in the reach-to-grasp task All conventions are as in **(B)**. Visuomotor encoding of the objects in object presentation **(D)** changes to motor encoding in the reach-to-grasp execution **(E)**. Adapted from Fattori et al. (2012), licensed under CC BY-NC-SA 4.0; Breveglieri et al. (2018), with permission from Oxford University Press.

Visual sensitivity of V6A to 3D object shapes was tested by presenting different objects of different shapes, without giving the monkey the possibility to reach and grasp them, because of a barrier placed between the hand and the object (Fattori et al., 2012). The monkey was rewarded for simply fixating the object, presented one at a time, always in the same spatial location, straight ahead. The 5 different objects are sketched at the top of Figure 5A. The responses of an example neuron are shown below. This cell showed a strong tuning for the object shape, displaying the maximum visual response to the presentation of the handle, an intermediate visual response to the presentation of the ball, plate, and ring, and no visual response to the presentation of the small cylinder in a groove. Note that all the objects appeared in the same part of the visual field (lower hemifield along the vertical meridian), so the differences in responses cannot be ascribed to the different parts of the visual field being stimulated (Fattori et al., 2012). To study how the entire pool of V6A neurons encoded the presentation of the different objects, we performed a hierarchical cluster analysis, the result of which is shown in Figure 5B. Objects that evoke similar responses in different neurons are placed close together. Despite the limited number of objects tested, a trend in the clustering can be appreciated: the ring and the ball are placed very close to one another and form a tight cluster, and the plate and the handle are clustered similarly. Then, these 2 clusters merge and group with the remaining object, the stick-in-groove. It seems that the neural discharges in V6A encode the form of the objects following a visual code, forming three different clusters: round objects, flat objects, and complex shapes.

We checked whether the object encoding observed in a task that involved only object fixation was present in V6A when the same objects were presented at the beginning of a reach-to-grasp action, i.e., in a visuomotor context. The example visuomotor neuron in Figure 5C selectively encoded both the object presented to the animal at the beginning of the trial (first peak, within the red box) and the grip used to grasp it (second peak of activation, within the blue box). In the case of the neuron shown in Figure 5C, both the presentation of the ball and the execution of the whole hand prehension evoked the maximum activation, while the presentation of the plate and its grasping evoked the weakest response. It must be noticed that the period highlighted in red is the only one in which there is visual stimulation, as all the remaining parts of the trial took place in complete darkness, apart from the fixation point which was barely visible and was used to help the monkey to keep its gaze stable and straight ahead. Figures 5D, E show the hierarchical cluster analyses performed on the visual (red) and the motor (blue) epochs for all the visuomotor cells recorded from V6A during a reach-to-grasp task (Fattori et al., 2012). It is evident that the clustering of the 2 epochs (vision before grasping and grasping) are different. In the visual epoch, the hierarchical cluster analysis reveals that the objects with a hole (ring and handle) are separated from the solid objects (ball, stick-in-groove, plate). It is likely that this clustering reflects the animal’s visuomotor behavior, as objects requiring a finger to be inserted into a hole in order to grasp them are grouped separately from those grasped by wrapping the fingers around them. We saw a visuomotor rule in this clustering.

We checked whether this type of object encoding remained stable throughout the reach-to-grasp trial (shown in Figure 5C), or whether it changed from object presentation to grasp execution. We found that the visuomotor neurons dynamically changed their encoding over the course of the trial. During movement execution (Figure 5E), the hook grip (ring) and the precision grip (plate, stick-in-groove) clusters are very close in the encoding space, probably due to the essential involvement of the index finger in grasping these objects. On the contrary, the finger prehension (handle) and whole-hand prehension (ball) are at a large distance from the other grips, and they are grasped without the index finger. These clusters found in the movement period rely on the use of the index finger for grasping, suggesting the existence of a motor code in V6A, with a transition from object encoding during object presentation, to motor encoding during movement execution in the dark. This highlights the plasticity of visual encoding along the course of the grasping trial. Moreover, the encoding patterns observed during object presentation in the reach-to-grasp task during object presentation (before the grasping) are different compared to the encoding done during object presentation in the simple visual fixation trial. A comparison of the clustering patterns in Figure 5B with those shown in Figure 5D highlights the different visual encoding within and outside of the grasping context. V6A shows complex discharges to objects according to the context.

## Encoding of object affordance

**FIGURE 6:**
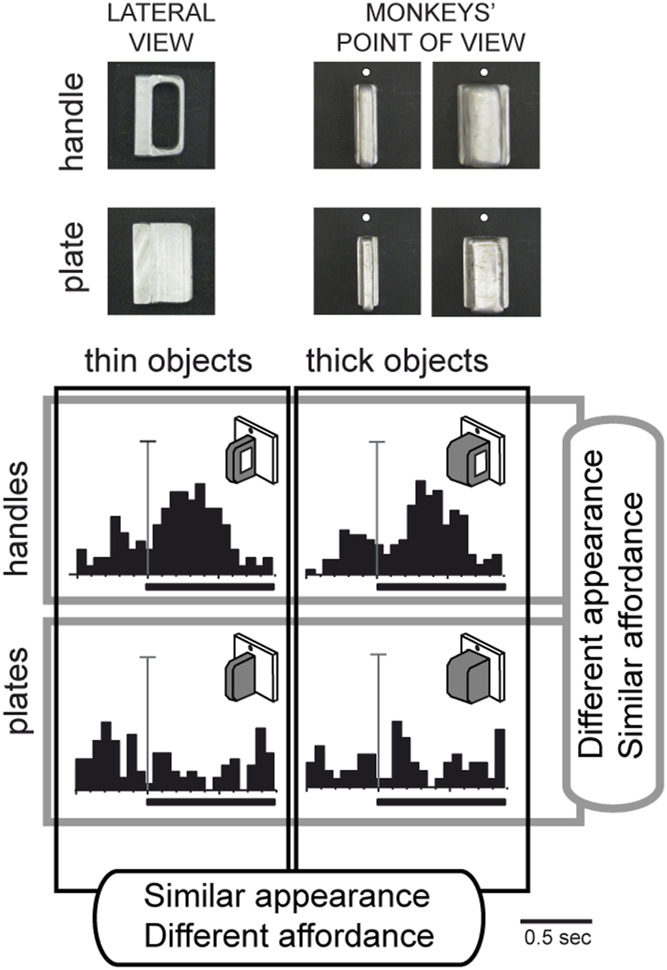
Vision of similar objects: encoding of affordance in V6A. Top, Photos of the objects used in the task (thin and thick handles and plates) as seen laterally and from the monkeys’ point of view. Bottom, example neuron tested for same/different affordance and same/different visual features. Activity is shown as peristimulus time histograms, aligned (long vertical line) with the onset of the object illumination (thick black line: time of object illumination). Vertical scale bars on histograms: 45 spikes/s. Top: response to handle; bottom: response to plate. Left, responses to thin versions of the objects. Right: responses to thick versions of the same objects. Very different visual features do not evoke different neural responses, but different affordances do. Adapted from Breveglieri et al. (2015), with permission from The MIT Press.

Given the involvement of area V6A in the control of prehension, we wondered whether, in addition to the object shape, V6A neurons were able to encode the affordance of an object to be grasped, i.e., the processing of critical visual features that evoke the potential actions that the subject can perform during the interaction with the object (Gibson, 1979). To address this question, we conducted a specific experiment (Breveglieri et al., 2015) in which the monkey looked at and then grasped a thin plate or a thin handle which were visually very similar from the monkey’s point of view, as shown at the top left of Figure 6. Despite the visual similarity, the monkey was able to distinguish the plate from the handle, shaping its hand correctly according to the object to be grasped, and using two different grips to grasp the plate and the handle. An example neuron response to the presentation of these 2 objects is shown at the bottom left of Figure 6, revealing a strong preference for the handle, while showing no response to the observation of the plate, despite its similar appearance. This suggests that the neuron encodes the affordance of the object rather than its visual attributes. This hypothesis was further supported when a thick version of the plate and handle were presented to the monkey (Figure 6, right side). The cell, again, discharged strongly to the presentation of the handle and poorly to the plate. Comparing the 4 discharges shown in Figure 6, it is evident that the neuron discharged equally well to the 2 handles, whether the thick or thin version, and showed no response to either of the plates. In other words, the discharge of this neuron during object presentation encoded the kind of action to be performed (i.e., the affordance, according to Gibson, 1979) and not the visual shape of the object, as typically observed in the ventral stream areas.

These data suggest that area V6A has access to feature information and uses it in a behaviorally relevant manner, modulating this information according to task demands. The apparent visual selectivity found in V6A may serve in the rapid transformation of visual representations into object-specific motor programs that is useful in visually guided grasping.

## Object encoding by mirror neurons in V6A

**FIGURE 7:**
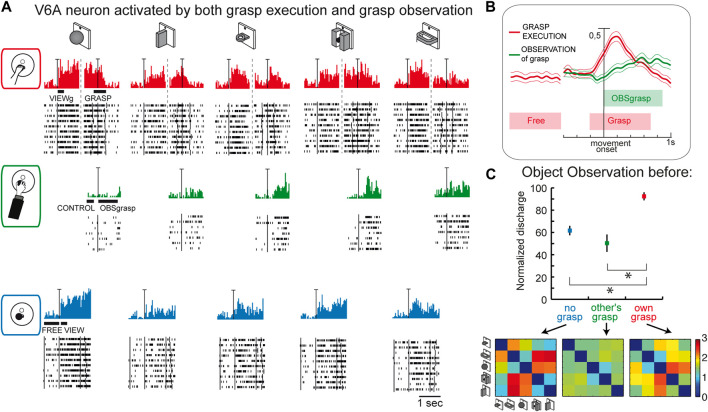
Mirror neurons in area V6A. **(A)** Response of a V6A mirror neuron during the Reach-to-grasp task in the light (top), during observation of another’s grasping action (center), and during observation of the object without grasping (bottom). The vertical scale bar on the histograms indicates 70 spikes/s. Bars below histograms indicate the duration of the epochs used for statistical analyses. **(B)**, Population discharges, expressed as averaged spike density functions, where continuous lines indicate the average neural activity recorded during grasping in the light, and dashed lines indicate the activity during the observation of the experimenter’s grasping. The thinner lines indicate the variability band (standard error of the mean). The neural discharge during the grasp observation showed a different time course compared to that evoked by the subject’s own action because of the longer time course of the actions performed by the experimenter. The vertical scale indicates 100% of normalized activity. **(C)**, Top: normalized average activity of mirror neurons during object observation in three different conditions: passive observation without grasp (no grasp; epoch VIEW, in blue), before the other’s grasp (epoch CONTROL, in green), and before the subject’s own grasp (epoch VIEWg, in red). Asterisks indicate significant differences. Error bars represent the standard error. Bottom: matrices showing the pairwise Euclidean distance between the population responses to two different objects during object observation in the three different conditions. These matrices were uncorrelated. Adapted from Breveglieri et al. (2019), with permission from Elsevier Science.

This last suggestion concerning the role of visual selectivity in V6A is further supported by the study investigating the mirror properties of its neurons (Breveglieri et al., 2019). As in the classic studies on mirror neurons (Gallese et al., 1996; Kraskov et al., 2009; Vigneswaran et al., 2013; Bonini et al., 2014; Mazurek et al., 2018), V6A neurons were tested during: i) the execution of reach-to-grasp actions by the animal (Figure 7A, top); ii) observation of the same actions performed by the experimenter (Figure 7A, center); iii) simple observation of the same objects outside the grasping context, with a barrier preventing any interaction with the object (Figure 7A, bottom). Some cells, as in the example reported in Figure 7A, show mirror properties by responding to both the animal’s and the experimenter’s grasping actions. Note that the mirror neurons found in V6A show limited congruence between discharges during execution and observation of the grasping action, differently from the original definition of mirror neurons (the “canonical” neurons of Gallese et al., 1996). This little or no correspondence in the discharge in V6A between the executed and observed actions is observable also in the discharge of the example cell shown in Figure 7A: the observation of the experimenter’s grasp (second row) evoked responses for three objects and these were not those eliciting the maximum activation during execution of the same actions (top). Indeed, in execution, the best discharge was for the ball, that did not evoke any discharge when grasped by the experimenter. The lack of congruence between action and observation in V6A is not so surprising because recently it has been found that even in ventral premotor cortex the proportions of congruent neurons is not different from chance (Papadourakis and Raos, 2019).

Considering the entire population of V6A mirror neurons (Figure 7B), the activity shows a peak during the execution of the grasping action and a later, but still significant, peak during the observation of the experimenter’s action.

A further analysis focusing on the visual presentation of the graspable objects shows that the visual encoding of the object in V6A changes according to the context, as summarized in Figure 7C. Here the neural discharge is analyzed around the time of object illumination in the 3 contexts (i.e., no grasp, other’s grasp, own grasp). Normalized population discharges differentiate between the different situations, with a clear preference for the object presentation before the occurrence of one’s own grasp (red). The confusion matrices obtained by comparing the activation during the presentation of 5 different objects in the 3 contexts (Figure 7C, bottom) are highly dissimilar, indicating a rather different representation by the same neural population in the different contexts in which the objects are presented. This indicates that the neural representation of an object in V6A changes according to the relevance of the object for the subject’s action.

In summary, all these data indicate that in V6A the neural representation of an object is context-dependent, influenced by whether grasping is allowed (and then performed), or whether the object is grasped by someone else. A plausible hypothesis is that the attentional load on the object could modulate neural activity, but further experiments are required to answer this question. These data suggest that object encoding in V6A is for action, but only if it is for one’s own movement. To activate V6A neurons it is necessary to know that the observed object will be the target of one’s own action, maybe with the purpose of better preparing the forthcoming action, or of allowing one to be ready to adjust the movement if something changes, or for other aspects related to visuomotor integration and action control.

## Discussion

The visual representations of the medial sectors of PPC seem to be linked to action control. The findings summarized here indicate that this is the case at least for an area of the PPC, area V6A, which has been extensively studied in the monkey in the past few decades. Despite the current limitations in temporal and spatial resolution of the non-invasive techniques available to study human brain functions, recent studies have identified a homologue of monkey V6A in humans (Cavina-Pratesi et al., 2010; Vesia et al., 2010; Galati et al., 2011; Gallivan et al., 2011; Monaco et al., 2011; Pitzalis et al., 2013; Pitzalis et al., 2015; Tosoni et al., 2015; Sulpizio et al., 2020; Sulpizio et al., 2023; Maltempo et al., 2021).

The single cell results reviewed here highlight how visual receptive fields of V6A cover a large part of the visual field (Galletti et al., 1999b), although the visual field representation is not point-to-point retinotopically organized, as it is in the nearby extrastriate visual areas. The representation of the lower visual hemifield is particularly emphasized. Interestingly, this part of the visual field shows psychophysical advantages for hand action control (Warman et al., 2023), both in the grasping (Brown et al., 2005) and in the pointing actions (Danckert and Goodale, 2001). This is in line with the presence, in V6A, of reaching and grasping neurons (Fattori et al., 2004; Fattori et al., 2005) and with the proposed functional role of V6A as a control area during prehension actions (Fattori et al., 2017; Galletti and Fattori, 2018).

The superior parietal lobule combines visual and somatosensory signals to monitor limb configuration (Graziano et al., 2000; Gamberini et al., 2018). The present review is particularly focused on determining whether and how the vision of the moving limb influences the activity of V6A neurons during arm movements. By playing with the illumination in which actions occur, thus adding or not adding visual information to proprioceptive/motor-related information, a possible role of the dorsomedial visual stream is highlighted, in integrating visual and motor signals to monitor and correct reaching and grasping actions. Altogether these results point to V6A playing a role as a state estimator in the circuits involved in planning and correctly executing reaching and reach-to-grasp movements (for review Grafton, 2010; Shadmehr et al., 2010; Fattori et al., 2017; Medendorp and Heed, 2019). Visual and somatosensory/motor-related inputs in V6A could allow this area to act as a comparator between the expected state of the movement, and the visual/somatosensory feedback evoked by the movement itself. From this comparison an error signal could stem that provides feedback to the frontal motor cortices, thus allowing movements to be corrected. Vision of the moving limb and of the environment in which the action unfolds might help V6A neurons to compare anticipated and actual sensory feedback evoked by the action itself and to evaluate the current state of the world and body that relates to choosing and specifying the most appropriate actions. Possible discrepancies between anticipated and actual sensory feedbacks may be signaled by V6A and be used to adjust the motor plan, so that the ongoing movement keeps in register with the desired one, resulting in an accurate reaching or reach-to-grasp movement. The co-presence of signals related to sensory feedback and predictive signals of future action has been recently found in the SPL (Filippini et al., 2022), confirming the potential role of this cortical region as a comparator between expected and actually executed movements. Indeed, the error signal may be extremely useful to premotor areas, that have a direct input from V6A (Matelli et al., 1998; Galletti et al., 2004; Gamberini et al., 2009; 2021) when orchestrating successful motor control. These neuronal properties representing differences in the degree to which cells are influenced by feedback control *versus* feedforward planning of reaching and reach-to-grasp find a parallel in the human brain with recent transcranial magnetic interference with V6A activity during reaching and grasping (Breveglieri et al., 2021; Breveglieri et al., 2022; Breveglieri et al., 2023), showing a causal involvement of human V6A in state estimation for reaching and grasping.

Regarding object vision, cognitive recognition is a typical function associated with the ventral stream (Ungerleider and Mishkin, 1982; Goodale and Milner, 1992), whereas the dorsolateral visual stream is shown to be able to process shape to correctly shape the hand during grasping (Romero et al., 2014; Theys et al., 2015; Schaffelhofer and Scherberger, 2016). We also reported that area V6A (part of the dorsomedial visual stream) shows visual responses tuned for objects. What is intriguing is that these visual properties seemed to be related to the object affordance (Breveglieri et al., 2015). Thus, we argue that, in the dorsomedial visual stream, area V6A codes for the reaching of motor components, but may also encode object shapes to plan and adjust visually guided grasping.

We also reported that visual responses in V6A are strongly tuned for objects, depending on the context in which the objects are presented: the encoding changes if the same objects are presented inside or outside the grasping context (Fattori et al., 2012). Contextual information regarding graspable objects may be processed in dorsomedial area V6A in order to guide interaction with objects, in cooperation with the dorsolateral area AIP to select or generate appropriate grasp movements, or in cooperation with ventral stream areas to identify actions based on objects (Wurm and Caramazza, 2022). Moreover, as shown by the mirror neuron study, the encoding is different according to whether the objects are targets of one’s own action or of another agent’s action, or are not action targets at all (Breveglieri et al., 2019). We suggest that the visual response of V6A mirror neurons to the presentation of an object depends on the pragmatic value of the object for the observer. V6A mirror neurons may participate in different computations in a flexible way: monitoring one’s own behavior or switching to a separate predictive activity when another agent is performing the action. The object encoding in this case may represent the motivational value of an action, coded at different levels of attention, according to the agent of the action. The object encoding by V6A mirror neurons also point toward a role of V6A in the online monitoring of one’s own actions.

All the visual functions studied so far in the medial parietal area V6A point to an encoding of the visual attributes/affordance of objects that are the target of our actions and that shape the action itself, together with the neural encoding performed in real time when the action unfolds. From the results reviewed here, it is evident that the contribution of V6A neurons may be called into play any time we interact with objects in the environment, or explore an environment to decide what to do there, and in any case of positive and fruitful interaction with the world. However, the knowledge of these processes is far from being complete, and this makes future research in this field challenging and motivating.
